# COVID-19 Vaccine Donations—Vaccine Empathy or Vaccine Diplomacy? A Narrative Literature Review

**DOI:** 10.3390/vaccines9091024

**Published:** 2021-09-15

**Authors:** Zhaohui Su, Dean McDonnell, Xiaoshan Li, Bindi Bennett, Sabina Šegalo, Jaffar Abbas, Ali Cheshmehzangi, Yu-Tao Xiang

**Affiliations:** 1 Center on Smart and Connected Health Technologies, Mays Cancer Center, School of Nursing, UT Health San Antonio, San Antonio, TX 78229, USA; szh@utexas.edu; 2 Department of Humanities, Institute of Technology, R93 V960 Carlow, Ireland; dean.mcdonnell@itcarlow.ie; 3 Program of Public Relations and Advertising, Beijing Normal University-Hong Kong Baptist University United International College, Hong Kong 519087, China; xiaoshan.li@utexas.edu; 4 University of the Sunshine Coast, Maroochydore, QLD 4558, Australia; bbennet1@usc.edu.au; 5 Department of Microbiology, Faculty of Medicine, University of Sarajevo, 71000 Sarajevo, Bosnia and Herzegovina; sabina.segalo11@gmail.com; 6 School of Media and Communication, Antai College of Economics and Management, Shanghai Jiao Tong University, Shanghai 200240, China; dr.abbas.jaffar@outlook.com; 7 Department of Architecture and Built Environment, Faculty of Science and Engineering, University of Nottingham Ningbo China, Ningbo 315100, China; Ali.Cheshmehzangi@nottingham.edu.cn; 8 Network for Education and Research on Peace and Sustainability (NERPS), Hiroshima University, Hiroshima 739-8527, Japan; 9 Centre for Cognitive and Brain Sciences, Department of Public Health and Medicinal Administration, Faculty of Health Sciences, Institute of Translational Medicine, Institute of Advanced Studies in Humanities and Social Sciences, University of Macau, Macao 999078, China

**Keywords:** COVID-19, vaccination, inequality, vaccine diplomacy, vaccine empathy

## Abstract

**Introduction:** Vaccine inequality inflames the COVID-19 pandemic. Ensuring equitable immunization, vaccine empathy is needed to boost vaccine donations among capable countries. However, damaging narratives built around vaccine donations such as “vaccine diplomacy” could undermine nations’ willingness to donate their vaccines, which, in turn, further exacerbate global vaccine inequality. However, while discussions on vaccine diplomacy are on the rise, there is limited research related to vaccine diplomacy, especially in terms of its characteristics and effects on vaccine distribution vis-à-vis vaccine empathy. Thus, to bridge the research gap, this study aims to examine the defining attributes of vaccine diplomacy and its potential effects on COVID-19 immunization, particularly in light of vaccine empathy. **Methods:** A narrative review was conducted to shed light on vaccine diplomacy’s defining attributes and effects in the context of COVID-19 vaccine distribution and dissemination. Databases such as PubMed and Medline were utilized for literature search. Additionally, to ensure up-to-date insights are included in the review, validated reports and reverse tracing of eligible articles’ reference lists in Google Scholar have also been conducted to locate relevant records. **Results:** Vaccine empathy is an individual or a nation’s capability to sympathize with other individuals or nations’ vaccine wants and needs, whereas vaccine diplomacy is a nation’s vaccine efforts that aim to build mutually beneficial relationships with other nations ultimately. Our findings show that while both vaccine empathy and vaccine diplomacy have their strengths and weaknesses, they all have great potential to improve vaccine equality, particularly amid fast-developing and ever-evolving global health crises such as COVID-19. Furthermore, analyses show that, compared to vaccine empathy, vaccine diplomacy might be a more sustainable solution to improve vaccine donations mainly because of its deeper and stronger roots in multilateral collaboration and cooperation. **Conclusion:** Similar to penicillin, automated external defibrillators, or safety belts amid a roaring global health disaster, COVID-19 vaccines are, essentially, life-saving consumer health products that should be available to those who need them. Though man-made and complicated, vaccine inequality is nonetheless a solvable issue—gaps in vaccine distribution and dissemination can be effectively addressed by timely vaccine donations. Overall, our study underscores the instrumental and indispensable role of vaccine diplomacy in addressing the vaccine inequality issue amid the COVID-19 pandemic and its potentials for making even greater contributions in forging global solidarity amid international health emergencies. Future research could investigate approaches that could further inspire and improve vaccine donations among capable nations at a global scale to advance vaccine equity further.

## 1. Introduction

Vaccine distribution is often unequal [1]. The answer to who should have nonessentials, such as the best piano in the world, could be reasonably varied, ranging from the richest, the luckiest, or the most interested to the best pianist. The answer to who should have essentials such as the coronavirus disease 2019 (COVID-19) vaccines in the wake of a fast-deteriorating global pandemic, on the other hand, should be simple and straightforward; those who need COVID-19 vaccination the most. Similar to penicillin, automated external defibrillators, or safety belts, COVID-19 vaccines are, essentially, life-saving consumer health technologies [2] or products that should be available to those who need them in a timely fashion. This is particularly true in light of the ever-evolving COVID-19 mutations, such as the Delta variant [3], which are situations that further highlight why vaccinations remain the most straightforward path into a post-pandemic reality [4]. Then comes the rub: across the globe, especially in poorer countries [5], why is it that the world’s most vulnerable communities to the pandemic—the old, the immunocompromised, and the frontline workers [6,7], are not given the vaccines they urgently needed to fend off COVID-19 infections and deaths?

### 1.1. Vaccine Availability Equals to Vaccine Accessibility, Only for the Global North

It is important to understand that global vaccine production has accelerated tremendously since it first became available—more than 12 billion vaccine doses will be produced in 2021 alone [1]. Yet ironically, or perhaps unsurprisingly, most of the available vaccines went to rich countries—as of 7 May 2021, high-income countries have grabbed approximately 5 billion COVID-19 vaccines, while on the other hand, low-income nations only managed to secure around 270 million doses [1]. Vaccine inequality has already been translated into health inequality; many thanks to a considerable surplus of COVID-19 vaccines, while North America has already vaccinated 30.57% of its population, followed by Europe (23.28%) and South America (12.82%), whereas continents, such as Asia and Africa, only have 4.48% and 1.01% of their populations vaccinated against the virus, respectively [7]. In other words, as rich countries such as the United States (U.S.) and Canada hoard doses up to five times their respective populations and counting [1], the Global South, countries ranging from India, Brazil, and Peru to Rwanda are still struggling and scrambling to secure vaccines to not only protect its most vulnerable from COVID-19 infections and deaths but also to prevent humanitarian crises from further deterioration [8]. Peru, for instance, although it has been shouldering a grim excess death rate (over-the-historical-average death toll) that is twice that of the U.S. [8], as of 7 May 2021, it only had 3.77% of its population vaccinated compared to 44.69% COVID-19 vaccinations in the U.S. [7]. These sobering statistics indicate that, while vaccine inequality is on the rise, vaccine empathy is in short supply.

### 1.2. The Humanitarian Imperative of Vaccine Empathy

Though successful COVID-19 vaccination campaigns are contingent on many factors ranging from effective vaccine communication, vaccine efficacy, vaccine distribution, vaccine administration, to equitable vaccine accessibility [9,10,11], among all these factors, vaccine accessibility is perhaps the most important contributor upon which the rest of the components depend. For instance, debates about which vaccine is more efficacious or easier to transport are meaningless unless people have access to actual COVID-19 shots so that they can test their efficacy or transport them between places. Arguably, the most effective approach to bridging vaccine inequality, measured by the time needed for enabling those who want vaccines access to COVID-19 shots, is via vaccine sharing mechanisms, such as vaccines donated by the World Health Organization’s (WHO) COVAX program [12]. Compared to other possible solutions to address the ever-widening vaccine inequality gap, ranging from waiving vaccine patents to building vaccine factories, vaccine donation is perhaps the most effective, efficient, and pressure-free alternative for vaccine have-nots to have access to COVID-19 vaccines in a timely fashion [13]. The time saved from pricing, payment, patent, manufacturing, delivery, administration, data procurement, along with the accompanying contractual negotiations, for instance, could translate into lives saved amid the continuing rampant pandemic; during the first week of May 2021, for instance, each hour, an average of 153 people died of COVID-19 in India alone [8].

It is important to note that, aiming to bridge vaccine inequality, countries across the world have been sharing their doses with those in need, especially nations equipped with vaccine development and production capabilities such as China, India, and Russia [14]. As of 5th May, outside of the COVAX vaccine distribution scheme, only five countries have made larger-than-100,000 donations to the vaccine have-nots: China (13.4 million), India (10.5 million), Turkey (190,000), Russia (158,000), and United Arab Emirates (UAE) (120,000) [14]. Unfortunately, none of the top vaccine donor countries are rich western countries, including nations that have been hoarding COVID-19 vaccines from the get-go [1]. Naturally, particularly in light of distribution issues identified in the WHO’s COVAX program (e.g., extremely slow roll-out) [15], a more hopeful alternative is for capable countries to show their vaccine empathy by sharing their vaccine surpluses with those in dire need. However, in light of the rampant vaccine nationalism sentiments [16] and the fact that COVID-19 herd immunity is difficult to achieve, particularly in light of vaccine hesitancy [10] and virulent mutations [17], it might become increasingly difficult for rich countries to share their “parked” COVID-19 doses [18].

The lack of vaccine donors might be further exacerbated by the toxic narratives that surround these vaccine donations, as countries that made the most COVID-19 donations are often referred to as “vaccine diplomats”, undermining the humanitarian significance of sharing possibly the most precious and life-saving properties amid the pandemic—COVID-19 vaccines. In other words, rather than seeing vaccine donations as a display of vaccine empathy, by fixating on the possible reciprocal favors that vaccine donors may or may not receive in the future, many scholars frame these donations as purely a transaction in the scheme of vaccine diplomacy [19,20,21,22]. Yet interestingly, while discussions on vaccine diplomacy are on the rise, there is a shortage of insights on vaccine diplomacy and how it might similar to or differ from vaccine empathy. Thus, to bridge the research gap, this study aims to examine the characteristics and effects of vaccine diplomacy in the context of the pandemic, particularly in light of COVID-19 vaccine empathy.

## 2. Methods

A narrative literature review was conducted to identify the characteristics and effects of vaccine diplomacy in the context of COVID-19, vis-à-vis vaccine empathy amid the pandemic. The narrative review method was chosen because it is a powerful tool to help: (1) “identify what has been accomplished previously, allowing for consolidation, for building on previous work, for summation, for avoiding duplication and for identifying omissions or gaps” in a relatively nascent field that only limited research is expected to have conducted and published [23] and (2) build a conceptual understanding of the defining attributes of vaccine diplomacy based on the characteristics and effects identified [24]. We reviewed two representative databases, PubMed and Medline, for potentially eligible articles, using keywords that are focusing on two themes: vaccine diplomacy and the COVID-19 pandemic. The search terms used for PubMed and Medline could be found in Table 1. In addition, ensuring that up-to-date insights are included in this review, validated reports and reverse tracing of eligible articles’ reference lists in Google Scholar have also been conducted to locate additional relevant records.

### Inclusion and Exclusion Criteria

The full list of the inclusion criteria we adopted to screen articles could be found in Table 2. Overall, we excluded records if they are: (1) not written in English, (2) not focusing on vaccine-related diplomacy, (3) not centering on the COVID-19 vaccine, and (4) not providing detailed information on the attributes or effects of vaccine diplomacy in the context of COVID-19.

## 3. Results

The database search was conducted on 10 May 2021. Only articles published between January 2020 to May 2021 were considered. After removing duplicates and screening the remaining records against the inclusion and exclusion criteria, 12 articles were selected for the final review and analysis. Key characteristics of these articles can be found in Table 3. In the following section, we will elaborate on study findings as well as their implications for future vaccine practices amid COVID-19 and beyond.

## 4. Discussion

Vaccine inequality undermines the speed, solidarity, and significance of society’s collective fight against the COVID-19 pandemic [34]. Though vaccine donations have the potential to bridge vaccine inequality across the globe effectively and efficiently, these timely rescues are often negatively referred to as and narrowly summed into “vaccine diplomacy”, often without offering a definition of the term [35,36,37]. However, particularly in light of vaccine nationalism [38], while the use and abuse of the term vaccine diplomacy could harm global vaccine collaborations (e.g., vaccine donations and vaccine loans [39]), there is a dearth of research in the literature. Thus, to address the research gap, this study sets out to investigate the characteristics and effects of vaccine diplomacy in the context of COVID-19 vaccine distribution. Specifically, we aim to find out the defining attributes of vaccine diplomacy vis-à-vis those of vaccine empathy and both practices’ potential effects on addressing vaccine inequality.

**Figure 1:**
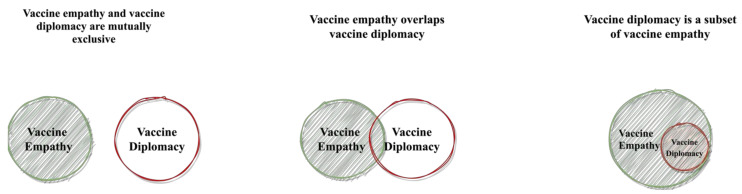
Paradigms for relationships between vaccine empathy and vaccine diplomacy.

Overall, our results suggest that while vaccine diplomacy and vaccine empathy have their strengths and weaknesses, they both have substantial potential to improve vaccine equity. Diplomacy is “the art of conducting relationships for gain without conflict [40]”. Vaccine diplomacy, in the context of this study, could be understood as a nation’s vaccine efforts that aim to build mutually beneficial relationships with other nations. Empathy, on the other hand, is “noticing another person’s feelings, making an inference of the mental state of another, and responding appropriately to that person’s state of mind [41]”. Vaccine empathy, in turn, could be understood as an individual or a nation’s capability to sympathize with other individuals or nations’ vaccine wants and needs. It is important to note that while the relationship between vaccine empathy and vaccine diplomacy could be mutually exclusive, it is also possible that vaccine diplomacy is a subset of vaccine empathy. An illustration of the possible relationships between these two concepts could be found in Figure 1.

However, vaccine donations, regardless of whether they are a result of vaccine empathy or vaccine diplomacy, could have a positive impact on the ever-increasingly widening vaccine inequality. Interestingly, the results of our narrative review suggest that, different from the makeup of news reports or other insights in mass media [35,36,37], the majority of scholarly articles on vaccine diplomacy published amid the pandemic supported the practice [18,19,25,26,27,28,29,31,32,33] with only two [22,30] of the twelve records expressing concerns about potential domestic backlash for donating vaccines. Our findings suggest that what WHO officials indicated on 11 May 2021, when they state that “vaccine diplomacy is not cooperation” and only “clear and clean cooperation” could yield benefits to pandemic prevention and control [42], might be in direct contrast to most academics’ positions on vaccine diplomacy [18,19,25,26,27,28,29,31,32,33]. While some criticisms towards vaccine diplomacy seem to be not completely unfounded, as the scale and severity of COVID-19 might make any potential future expected gains associated with these donations seem uncaring, possibly bordering on unsympathetic [19], the significance of the potential sustainability of vaccine diplomacy, which has deeper and stronger roots in multilateral cooperation and collaboration compared with vaccine empathy, along with how this sustainability might impact COVID-19 vaccine donations in the long run, should not be overlooked.

Arguably, though COVAX was spearheaded by the WHO, it is difficult to not consider countries that donated to the program as not practicing vaccine diplomacy, particularly in light of the presence of rotating media reports. It is also important to factor in the fact that vaccine donations can hardly be carried out by individuals, as opposed to nations—partially due to COVID-19 vaccines’ limited availability, high maintenance (e.g., reliance on sophisticated freezers), and unwavering demand [20,21]. These doses can hardly be donated anonymously or not in the form of “vaccine diplomacy”. These insights combined suggest that COVID-19 vaccine donations might almost always be associated with a nation donor, even under WHO’s COVAX program and in light of vaccine diplomacy. Detailed information on the defining attributes of vaccine empathy and vaccine diplomacy can be found in Table 4.

Our analyses indicate that, in contrast to vaccine diplomacy, vaccine empathy can exist independently of international collaborations and may not necessarily lead to actions or practices that improve vaccine equality. For instance, while many countries, including vaccine hoarders, such as the U.S. and Canada, might hold considerable vaccine empathy towards vaccine have-nots, due to factors such as lack of vaccine resources or domestic political pressure [18], they may not have the ability to act on their empathic emotions or thoughts. Findings further show that vaccine diplomacy could be a more effective and sustainable solution to address the issue of vaccine inequality, many thanks to its deeper roots in international collaboration and cooperation compared to vaccine empathy [43,44,45]. Furthermore, our results suggest that vaccine diplomacy’s sustainability could often translate into a continued commitment to vaccine donations, reciprocal relationships between the donor and recipient countries of the vaccines, and limited unintended consequences imposed on the recipient countries due to potential agreements, if not binding contracts, associated with the donations. In essence, ranging from short-term vaccine donations to long-term international collaborations, vaccine diplomacy reinforces positive actions that improve vaccine equality and reinforce global solidarity, which, in turn, provides much-needed help and humanity in a pandemic-beaten society.

It is important to note that although COVID-19 vaccine donor nations may expect future favors returned with their donations, these favors may or may not become realized in the future. In particular, due to lack of precedence, it is possible that what happens amid the once-in-a-century COVID-19 pandemic stays in the pandemic. Not to mention that countries that donate their vaccines, as we have seen from India’s example [46], are taking substantially greater risks compared to hoarder nations, such as becoming more vulnerable to potential future COVID-19 outbreaks and more prone to facing domestic backlash. In other words, considering the concrete vaccines donated, tangible inequality gaps bridged, and the positive sentiments associated with vaccine empathy displayed and delivered via doses shared, along with the possibility of no reciprocal favors returned post-pandemic and the considerable domestic pressures donor nations shoulder, greater acknowledgment, if not more encouragement, should be given to countries that endorse vaccine diplomacy. These insights combined suggest that, rather than pouring negative narratives to dampen the significance of bridging vaccine inequality via vaccine donations, it is perhaps more meaningful to appreciate and applaud both vaccine empathy and vaccine diplomacy in light of their results; improved COVID-19 vaccine equality, lives and livelihoods saved, as well as common humanity and global solidarity forged and reinforced.

## 5. Limitations

While this study fills important research gaps, it is not without limitations. First of all, our review is limited in scale and scope, which means that the insights provided in the analysis could be limited. Furthermore, the methodology we adopted is a narrative review approach, which, compared to methods such as systematic reviews, is limited in reproducibility and replicability [23].

## 6. Conclusions

Vaccine inequality, though man-made and complicated, is nonetheless a solvable issue—gaps in vaccine distribution and dissemination can be effectively addressed by timely vaccine donations that are enabled by vaccine diplomacy if not necessarily vaccine empathy. In this study, we examined the defining attributes of vaccine diplomacy and its potential effects on COVID-19 immunization in light of vaccine empathy. Albeit its shortcomings, the results underscore the instrumental and indispensable role of vaccine diplomacy in bridging the vaccine inequality issue amid the pandemic. Future research could investigate ways that could inspire and improve vaccine donations among vaccine-rich nations, especially countries that tend to hoard their vaccine surpluses, at a global scale to advance vaccine equity further so that society at large can start to build better together.

## Figures and Tables

**Table 1:** PubMed and Medline search strategy.

Theme	Search String
Vaccine Diplomacy	diplomacy(MeSH) OR diplomacy(TIAB) OR “health diplomacy” (MeSH) OR “health diplomacy” (TIAB) OR “medical diplomacy” (MeSH) OR “medical diplomacy”(TIAB) OR “vaccine diplomacy” (MeSH) OR “vaccine diplomacy” (TIAB) OR “science diplomacy” (MeSH) OR “science diplomacy” (TIAB)
COVID-19	((coronavirus OR “corona virus” OR coronavirinae OR coronaviridae OR betacoronavirus OR covid19 OR “covid 19” OR nCoV OR “CoV 2” OR CoV2 OR sarscov2 OR 2019nCoV OR “novel CoV” AND (“severe acute respiratory” OR pneumonia) AND (outbreak)) OR “Coronavirus”(Mesh) OR “Coronavirus Infections”(Mesh) OR “COVID-19” (Supplementary Concept) OR “severe acute respiratory syndrome coronavirus 2” (Supplementary Concept) OR “Betacoronavirus”(Mesh))

**Table 2:** Study inclusion criteria.

Data Type	Inclusion Criteria
Language	English
Study context	Vaccine diplomacy in the wake of COVID-19
Vaccine type	COVID-19 vaccines
Study design	Provides detailed information on the attributes and effects of vaccine diplomacy in the context of COVID-19

**Table 3:** Key articles included in the analysis.

Author	Year	Country *	Title	Policy Focus	Vaccine Diplomacy Position
AlKhaldi et al. [25]	2021	Canada	Rethinking and strengthening the Global Health Diplomacy through triangulated nexus between policy makers, scientists, and the community in light of the COVID-19 global crisis	Global	For
Bollyky et al. [26]	2021	U.S.	A year out: Addressing international impacts of the COVID-19 pandemic	U.S.	For
Chattu et al. [27]	2021	Canada	Global health diplomacy at the intersection of trade and health in the COVID-19 era	Global	For
Guidry et al. [18]	2021	U.S.	U.S. public support for COVID-19 vaccine donation to low- and middle-income countries during the COVID-19 pandemic	U.S.	For
Javed et al. [28]	2020	China	Strengthening the COVID-19 pandemic response, global leadership, and international cooperation through global health diplomacy	Global	For
Kobierecka et al. [19]	2021	Poland	Coronavirus diplomacy: Chinese medical assistance and its diplomatic implications	China	For
Lancet Commission on COVID-19 Vaccines and Therapeutics Task Force Members [29]	2021	U.K.	Operation Warp Speed: Implications for global vaccine security	Global	For
Pannu et al. [30]	2021	U.S.	The state inoculates: Vaccines as soft power	Global	Against
Sharun et al. [31]	2021	India	COVID-19 vaccine diplomacy and equitable access to vaccines amid ongoing pandemic	India	For
Sharun et al. [32]	2021	India	India’s role in COVID-19 vaccine diplomacy	India	For
Usher et al. [22]	2021	U.K.	Uncertainties over EU COVID-19 vaccine sharing scheme	EU	Against
Vanderslott et al. [33]	2020	U.K.	Health diplomacy across borders: The case of yellow fever and COVID-19	Global	For

**Note.** * Country refers to the first author’s affiliation location. EU: European Union; U.K.: the United Kingdom; U.S.: the United States.

**Table 4:** Definitions and defining characteristics of vaccine empathy and vaccine diplomacy.

	Empathy	Vaccine Empathy	Diplomacy	Vaccine Diplomacy
**Definition**	Empathy is “noticing another person’s feelings, making an inference of the mental state of another, and responding appropriately to that person’s state of mind.” [41]	Vaccine empathy is an individual or a nation’s capability to sympathize with other individuals or nations’ vaccine wants and needs.	Diplomacy is defined as “the art of conducting relationships for gain without conflict.” [40]	Vaccine diplomacy is a nation’s vaccine efforts that aim to build mutually beneficial relationships with other nations.
**Key Stakeholder**	Individuals and/or nations	Individuals and/or nations	Nations	Nations
**Defining Attribute**	People or nations act out of (vaccine) empathy are: Not self-interested Good-intentioned Guided by altruistic ideals (e.g., a healthy world) Possible ulterior motives (e.g., post-COVID-19 normalcy) May or may not have the ability to offer vaccine-related help to other individuals/nations Can be carried out independently/unilaterally		Nations act out of (vaccine) diplomacy considerations are: Self-interested Good-intentioned Possible altruistic ideals (e.g., a healthy world) Guided by ulterior motives (e.g., possible political favors) Have the talents and/or resources to offer vaccine-related help to other individuals/nations Cannot be carried out independently—have to be based on multilateral cooperation; Need the support of a multidisciplinary team that involves public health, law, management, etc.	
**Outcome**	Possible tangible help delivered No expected material gains Possible cognitive/emotional gains (e.g., improved self-worth) Possible backlash ○ From international stakeholders, if concerns are not translated into actions ○ From domestic stakeholders, for not focusing solely on national interests		Tangible help delivered Possible expected material gains Possible cognitive/emotional gains (e.g., improved self-worth) Possible backlash ○ From international stakeholders, for potential gains in soft power ○ From domestic stakeholders, for sharing vaccines to address international vaccine needs ○ From vaccine recipients, if the collaboration is inequitable	
**Strength & Shortcoming**	More likely to induce unintended consequences ○ Guided solely by the party’s perceived subjective reality, rather than mutually agreed understandings Less sustainable ○ The relationship is solely conditioned on one party’s interest and ability to provide help ○ The receiving party may refuse to accept empathic attention or help Unanticipated gains ○ Selfless acts could induce possible material and spiritual gains		Less likely to induce unintended consequences ○ Guided by the mutually agreed understandings More sustainable ○ Relationship is conditioned on two party’s mutually agreed interest and parameters Unanticipated losses ○ The other party may refuse to repay the favor ○ Potential domestic backlash ○ A nation’s failing ability to meet its promises (e.g., ill planning or COVID-19-caused vaccine crunch)	

## Data Availability

While this paper does not report raw data, all information highlighted can be sourced in the original papers published using the references below.

## Abbreviations

COVID-19	The coronavirus disease 2019
UAE	United Arab Emirates
U.S.	The United States
WHO	World Health Organization
